# Yeast surface display identifies a family of evasins from ticks with novel polyvalent CC chemokine-binding activities

**DOI:** 10.1038/s41598-017-04378-1

**Published:** 2017-06-27

**Authors:** Kamayani Singh, Graham Davies, Yara Alenazi, James R. O. Eaton, Akane Kawamura, Shoumo Bhattacharya

**Affiliations:** 1 RDM Division of Cardiovascular Medicine and Wellcome Trust Centre for Human Genetics, University of Oxford, Roosevelt Drive, Oxford, OX3 7BN United Kingdom; 2 Dept of Chemistry, University of Oxford, Mansfield Road, Oxford, OX1 3TA United Kingdom

## Abstract

Chemokines function via G-protein coupled receptors in a robust network to recruit immune cells to sites of inflammation. Due to the complexity of this network, targeting single chemokines or receptors has not been successful in inflammatory disease. Dog tick saliva contains polyvalent CC-chemokine binding peptides termed evasins 1 and 4, that efficiently disrupt the chemokine network in models of inflammatory disease. Here we develop yeast surface display as a tool for functionally identifying evasins, and use it to identify 10 novel polyvalent CC-chemokine binding evasin-like peptides from salivary transcriptomes of eight tick species in *Rhipicephalus* and *Amblyomma* genera. These evasins have unique binding profiles compared to evasins 1 and 4, targeting CCL2 and CCL13 in addition to other CC-chemokines. Evasin binding leads to neutralisation of chemokine function including that of complex chemokine mixtures, suggesting therapeutic efficacy in inflammatory disease. We propose that yeast surface display is a powerful approach to mine potential therapeutics from inter-species protein interactions that have arisen during evolution of parasitism in ticks.

## Introduction

Chemokines are secreted small extracellular proteins that are major drivers of inflammation in diverse diseases^1^. The 46 human chemokines fall into four groups - CC, CXC, XC and CX3C - defined by the spacing between N-terminal cysteine residues^2^. Structural features of chemokines include a flexible N-terminus, an invariant pair of disulphide bonded cysteine residues, a N-loop, a three-stranded β-sheet with loops designated 30S (between β1-β2) and 40S (between β2-β3), and a C-terminal helix. Chemokines function to recruit inflammatory and immune cells, including monocytes, neutrophils, eosinophils, dendritic cells, and T and B lymphocytes, to sites of inflammation and injury^3–5^. They bind to a family of G-protein coupled chemokine receptors (GPCRs) on these cells through two chemokine receptor sites (CRS1 and CRS2). The chemokine globular core binds to the extracellular N-terminus (CRS1) of the receptor via the proximal N-terminus and N-loop/40S loop grooves, whereas the chemokine distal N-terminus binds to CRS2 in the GPCR pocket, and is essential for activating signaling and chemotaxis^6^. In addition, chemokines bind with low affinity to glycosaminoglycans (GAGs) on the surface of endothelial cells, and this binding is necessary for chemokine function *in vivo*.

The chemokine system functions as a robust network. Properties of this network that engender robustness are the expression of multiple chemokine receptors on inflammatory cells^7^, expression of several chemokines in diseased tissues^8^, polyvalent chemokine-receptor interactions - with chemokines typically targeting more than one receptor, and receptors typically being activated by more than one chemokine^2^, synergistic and cooperative interactions between chemokines and chemokine receptors^9^, and feed-forward loops wherein inflammatory cells recruited to diseased tissue themselves secrete chemokines amplifying the network response^10^. The robustness of the chemokine network is clearly demonstrated by the observation that targeting individual chemokines or receptors has failed as a strategy to develop effective therapeutics for inflammatory disorders^7, 8, 11^.

A number of pathogens have evolved distinct peptides to target the chemokine network. These peptides have one common feature, namely, that they are typically polyvalent and target multiple chemokine network nodes (reviewed in ref. 11). Viral peptides such as MC148 and vMIP-II are homologous to chemokines, and bind and inhibit multiple chemokine receptors. Other viral proteins (CKBPs, chemokine binding proteins^12^) such as herpesvirus R17 and M3, papova virus CBP, and poxvirus CrmD and vCCI bind multiple chemokines typically via either the proximal N-terminus or via the N-loop/40s loop groove, preventing binding to CRS1^6^. A characteristic of CKBPs is that they target the invariant disulfide in all chemokines^13^.

Tick saliva contains proteins that suppress chemokine-driven inflammation by binding and neutralizing multiple chemokines simultaneously^14, 15^, helping them to suck blood for several weeks without eliciting inflammation^16^. Two related polyvalent CC-chemokine binding proteins, evasins 1 and 4, and one unrelated polyvalent CXC-binding protein, evasin 3, have been cloned from the brown dog tick *Rhiphicephalus sanguineus* ^15, 17^. The structure of evasin 1 in complex with the chemokine CCL3 shows that it has 4 intra-chain disulfide bonds, and targets the N-terminus of CCL3 in addition to the invariant disulfide^13, 18, 19^. *In silico* and mutagenesis studies indicate that evasin 4 also targets the N-terminus of CCL3^19^. Evasin 3 shows no sequence homology to evasins 1 or 4, and has a novel structural fold^15^.

Certain properties of evasins suggest that they may be potential therapeutics in inflammatory disease. Like other CKBPs, the ability of evasins to disrupt the chemokine network by binding multiple chemokines provides significant advantages over monoclonal antibodies that target single chemokines and have been unsuccessful as therapeutics for inflammation. The preferential binding to discrete subsets of chemokines could provide a method to target the disease-relevant chemokine network without unnecessarily targeting all chemokines. Evasins are highly glycosylated proteins, and as glycosylation is a well-recognized immune evasion strategy^20^, this predicts reduced immunogenicity^21^. Evasins 1 and 4 inhibit inflammation in a diverse range of pre-clinical animal models, including pancreatitis, joint inflammation, lung inflammation and fibrosis, post-myocardial infarction injury, psoriasis, and graft-versus-host disease (reviewed in ref. 21). Evasins administered subcutaneously have systemic anti-chemokine effects and do not appear to be significantly immunogenic in mice. This makes it likely that they have the potential, like other naturally occurring peptides such as coversin, a tick salivary ﻿peptide that targets complement, to be translated for clinical therapy^22^.

Bioinformatic analysis of salivary transcriptomes from species of ticks that are important human disease vectors indicates that they likely contain novel evasins^23, 24^. We speculated that such novel tick evasins may have distinct but selective chemokine binding characteristics that would make them valuable tool compounds and potential therapeutics for a broad range of inflammatory diseases. Here, we describe the development of yeast surface display for tick evasins, and the identification of a novel family of polyvalent CC chemokine-binding evasin peptides from tick species using this technology.

## Results

### Yeast surface display of evasins

**Figure 1:**
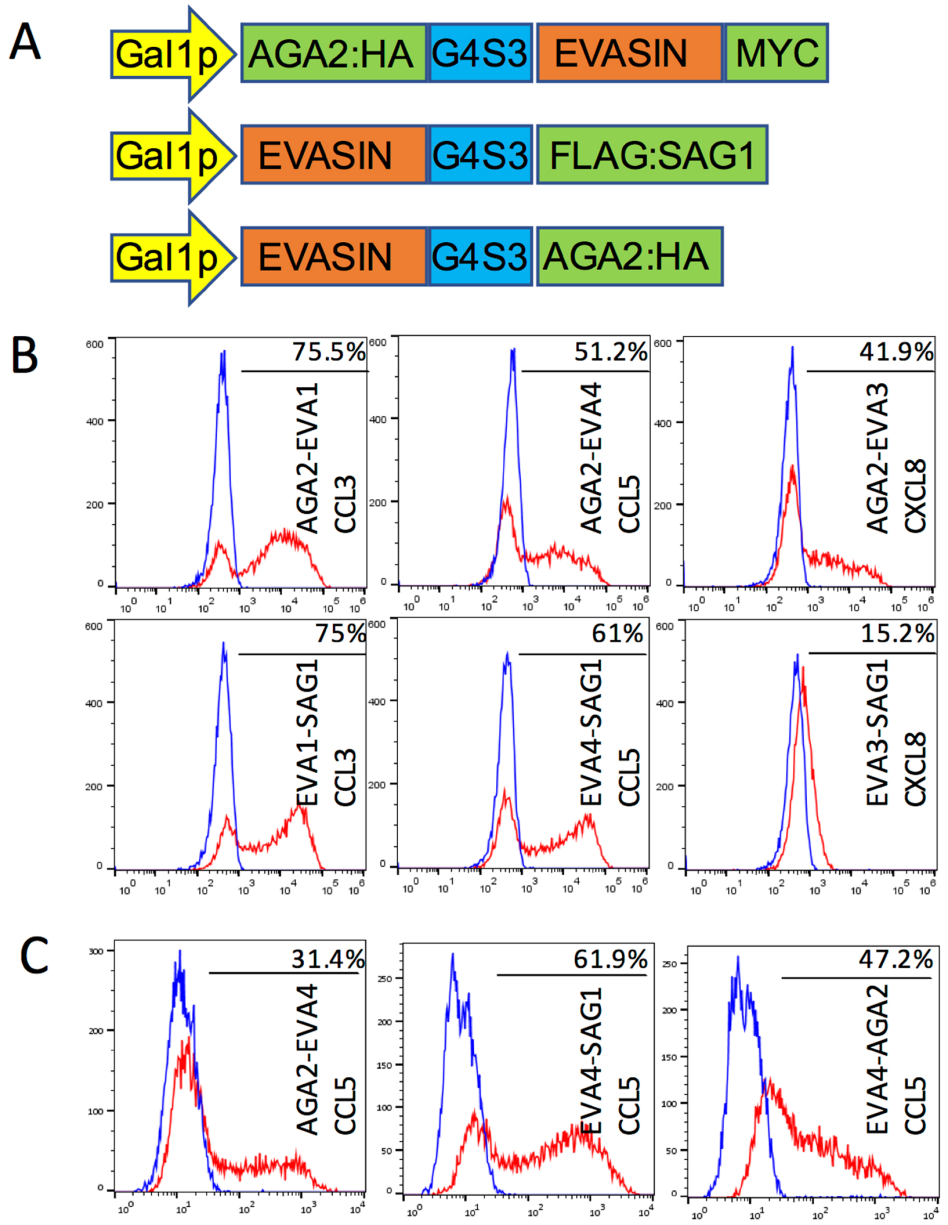
Yeast surface display of evasins. (**A**) Arrangement of evasin expression constructs (not to scale). Evasin fusion peptides were either tagged at the N-terminus with a AGA2 peptide (top), or at the C-terminus with SAG1 (middle) or AGA2 peptides (bottom). The position of antibody epitope tags (HA, MYC, FLAG), and the (Gly_4_Ser)_3_ linker (G4S3) is also indicated. Evasin fusion expression was driven by a Gal1p promoter. (**B**) Fluorescence profiles (red curves) of yeast displaying evasin 1 (left), evasin 4 (middle) and evasin 3 (right) panels incubated with indicated biotinylated chemokines and streptavidin-AF647(red curves). Data show a representative experiment of three biological repeats. Surface display tags were placed at the evasin N-terminus (AGA2, top panels) or at the evasin C-terminus (SAG1, bottom panels). Y-axis shows cell count (side scatter), and x-axis the fluorescence intensity on a log-scale. The blue curve is the profile of yeast containing a negative control surface display vector plasmid treated as above, and is used to determine background. The percentages of cells exceeding background levels in evasin displaying yeast are indicated. (**C**) Fluorescence profiles (red curves) of yeast displaying evasin 4 incubated with biotinylated CCL5 and streptavidin-AF647 (red curves). Data show a representative experiment of three biological repeats. Surface display tags were placed at the evasin N-terminus (AGA2, left panel) or at the evasin C-terminus (SAG1, AGA2, middle and right panels respectively). y-axis shows cell count, and x-axis the fluorescence intensity on a log-scale. The blue curve is the profile of yeast containing a negative control surface display vector plasmid treated as above, and is used to determine background. The percentages of cells exceeding background levels in evasin 4 displaying yeast are indicated.

To determine if functional evasins could be displayed on yeast surface, evasins 1, 3 and 4 were expressed with yeast surface display tags AGA2 at the N- or C-terminus^25, 26^, or SAG1 at the C-terminus^27^, under the control of a galactose inducible promoter (Fig. 1A). We used different yeast surface display methods as it was unclear if the nature or position of the tag would affect evasin structure or function as in the case of antibodies^26^. To minimize steric hindrance and loss of evasin function we inserted a (Gly_4_S)_3_ linker between evasins and surface display tags. Following galactose induction, yeast cells were labelled with a target biotinylated chemokine, followed by fluorescent streptavidin conjugate and analysed by flow cytometry. Yeast expressing evasins showed increased fluorescence compared to similarly treated controls (Fig. 1B,C). For evasins 1 and 4 the C-terminal fusions performed equivalently to the N-terminal fusion (Fig. 1B,C). For evasin 3 we noted that the N-terminal AGA2 tag performed significantly better (Fig. 1B). Consistent with observations from other yeast display methods^28^, we noted that a proportion of yeast do not display the protein. We assayed this using antibodies to epitope tags and found that ~60–80% of yeast express the display tag (see Supplementary Fig. S1). These results indicated that functionally active evasins with different structural folds can be expressed on yeast cell surface, and provided proof of concept that yeast surface display could be used to functionally characterize evasins. They also indicated that the location and nature of the display tag can affect evasin function.

### Identification of putative evasins by searching transcriptome datasets

Publicly available salivary transcriptome datasets from Prostriate (*Ixodes ricinus*) and Metastriate (*Amblyomma cajennense, A. maculatum, A. triste, A. parvum, A. americanum, Rhiphicephalus pulchellus, R. sanguineus*) ticks were initially searched with the sequences of evasins 1,3 and 4, and then with the sequences of functional novel evasins (P467_RHIPU and P546_AMBCA, identified below), using three iterations of psiBLAST. We identified 352 distantly related putative evasins from the eight tick species.

### Creation and screening of a yeast surface display mini-library

**Figure 2:**
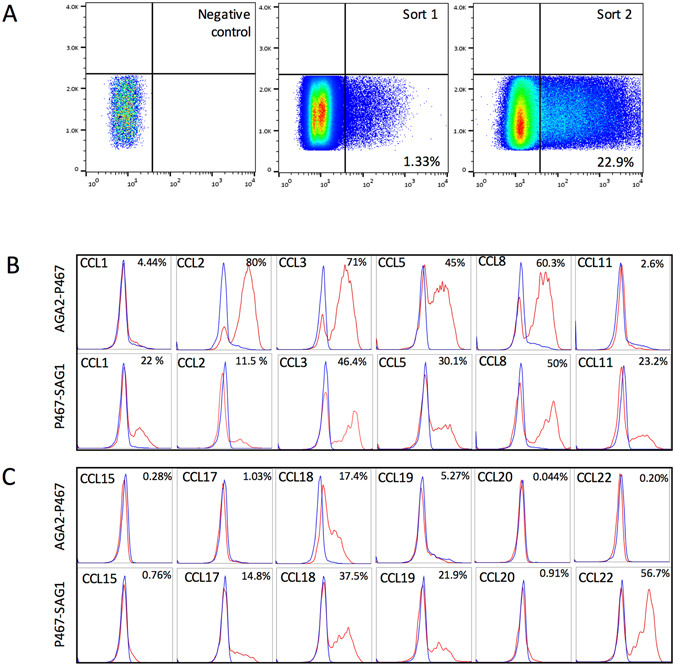
FACS screening of mini-library and retesting of recovered clones. (**A**) Fluorescence profiles of yeast surface display library incubated with streptavidin -AF647 alone (negative control), and incubated with biotinylated CCL5 plus streptavidin -AF647 (middle panel). The sorting gate was identified from the negative control, and was used to sort CCL5 binding yeast from the library. Yeast were sorted a second time similarly (right panel). Y-axis shows cell count and x-axis the fluorescence intensity on a log-scale. The proportions of cells within the sorting gate are indicated as a percentage. (**B,C**) Binding of P467_RHIPU to a panel of chemokines. Yeast surface display fluorescence profiles showing the binding of P467_RHIPU (N-terminal AGA2 fusion, top panels), and C-terminal SAG1 fusion) bottom panels, to a panel of CC chemokines as indicated in the figure. Yeast expressing P467_RHIPU are shown in red, and control yeast bearing empty vector surface display plasmid are shown in blue. Y-axis shows cell count, and x-axis the fluorescence intensity on a log-scale. The proportion of cells exceeding background are indicated as a percentage. The peptide sequence prefix indicates the identity, and suffix RHIPU indicates the species *Rhipicephalus pulchellus*.

To establish if yeast surface display could be used to identify novel functional evasins from a pool of putative evasins, we initially created a mini-library encoding 24 putative mature evasin peptides. These were fused to surface display tags at either the N-terminus (to AGA2) or at the C-terminus (to SAG1 or AGA2). Pooled libraries were transformed into yeast, cells labelled with biotinylated CCL5 followed by streptavidin-AF647 and then sorted by FACS to identify and recover a population of positive cells, using a gate determined by omitting the chemokine (Fig. 2A). Positive cells recovered in the first round were re-grown, and a second round of FACS was performed as above to further enrich the positive cell pool. This pool was plated at low density to enable picking of individual yeast clones. We initially selected for CCL5 binding evasins in these experiments as it is an important monocyte recruiting chemokine in cardiovascular disease^29^. Library plasmids from individual clones were rescued, sequenced to identify the peptide, and re-transformed to EBY100 yeast for re-testing by FACS (Fig. 2B,C). Four different clones were obtained, and we performed detailed retesting of one clone, P467_RHIPU, which was obtained in both N and C-terminally tagged orientations, against a panel of chemokines to examine its binding profile. This analysis showed that it clearly bound to CCL5, which it was initially selected for, as well as CCL2, CCL3, CCL8, CCL18 in the N-terminal AGA2 fusion. In the C-terminal SAG1 fusion, it revealed additional binding to CCL1, CCL11, CCL17, CCL19 and CCL22 (Fig. 2B,C). These results indicated that novel evasin clones could indeed be functionally recovered from a library of putative evasins using yeast surface display, and that a variety of chemokines could be used in such screens. They also confirmed that the nature and location of the surface display tag affected the chemokine-binding function of an evasin unpredictably, and that P467_RHIPU likely has two binding sites, as N and C-terminally tagged constructs bound different chemokines.

### Creation and screening of a yeast surface display maxi-library

We next constructed a maxi-library containing sequences encoding the mature peptides of 352 putative evasins identified by searching transcriptome databases. These were fused at the N-terminus to AGA2 or at the C-terminus to SAG1 or AGA2, and a pooled maxi-library created as above. We screened the maxi-library as described above using 13 of the 26 known human CC–chemokines that were commercially available as biotinylated versions. These were CCL1, CCL2, CCL3, CCL4, CCL5, CCL8, CCL11, CCL15, CCL17, CCL18, CCL20, CCL22 and CCL25. We performed screens with each biotinylated chemokine individually, and recovered 26 novel CC chemokine binding evasins. To date we have expressed, purified and characterized 10 of the 26 CC-chemokine binding evasins identified by yeast surface display and report them here (Table 1). Clones were obtained in either orientation, with a predominance of C-terminally tagged clones.

**Table 1:** Evasin clones recovered in human CC chemokine screens. The position of the surface display tag (clone tag) is described as NXCY, where X is the number of clones recovered with the tag at the N-terminus, and Y the number of clones recovered with the tag at the C-terminus. Abbreviations: MW – molecular weight (KDa), N-Glyc – predicted N-glycosylation sites, O-Glyc - predicted O-glycosylation sites. Length refers to number of amino acid residue.

Evasin	Accession	Clone Tag	Chemokine screen	Length	MW	pI	N-Glyc	O-Glyc
P991_AMBCA	JAC19654.1	N11C30	CCL2, CCL4, CCL5, CCL8, CCL17, CCL18, CCL19, CCL22	108	11.9	4.6	13, 33, 36, 50, 64, 72, 94	6, 7, 74
P985_AMBPA	JAC24842.1	N1C3	CCL5	108	11.7	4	21, 45, 50, 79, 87, 93	10, 23
P546_AMBCA	JAC18992.1	N1C7	CCL1, CCL3, CCL5, CCL22	97	11.1	5.2	24	3
P974_AMBCA	JAC18993.1	N6C35	CCL1, CCL3, CCL4, CCL8, CCL17, CCL18, CCL22	97	11.1	5.2	24	None predicted
P983_AMBCA		N3C11	CCL2, CCL3, CCL8	98	11	4.6	27, 46	None predicted
P1181_AMBMA	JAC19634.1	N1C2	CCL3, CCL4	90	10.1	4.7	20, 47, 78	None predicted
P1182_AMBMA	AEO33551.1	N4C12	CCL2, CCL3, CCL4, CCL8, CCL18	89	9.7	4.9	19, 46, 77	None predicted
P1183_AMBTR	JAC29608.1	N2C6	CCL2	90	10	4.6	20, 47, 78	None predicted
P1180_AMBTR	JAC29349.1	N6C31	CCL2, CCL3, CCL4, CCL8, CCL18	91	10.2	4.7	19, 46, 77	None predicted
P467_RHIPU	JAA60786.1	N6C5	CCL1, CCL2, CCL3, CCL5	106	11.5	4.6	28, 73	None predicted

### Sequence analysis of novel evasin peptides

**Figure 3:**
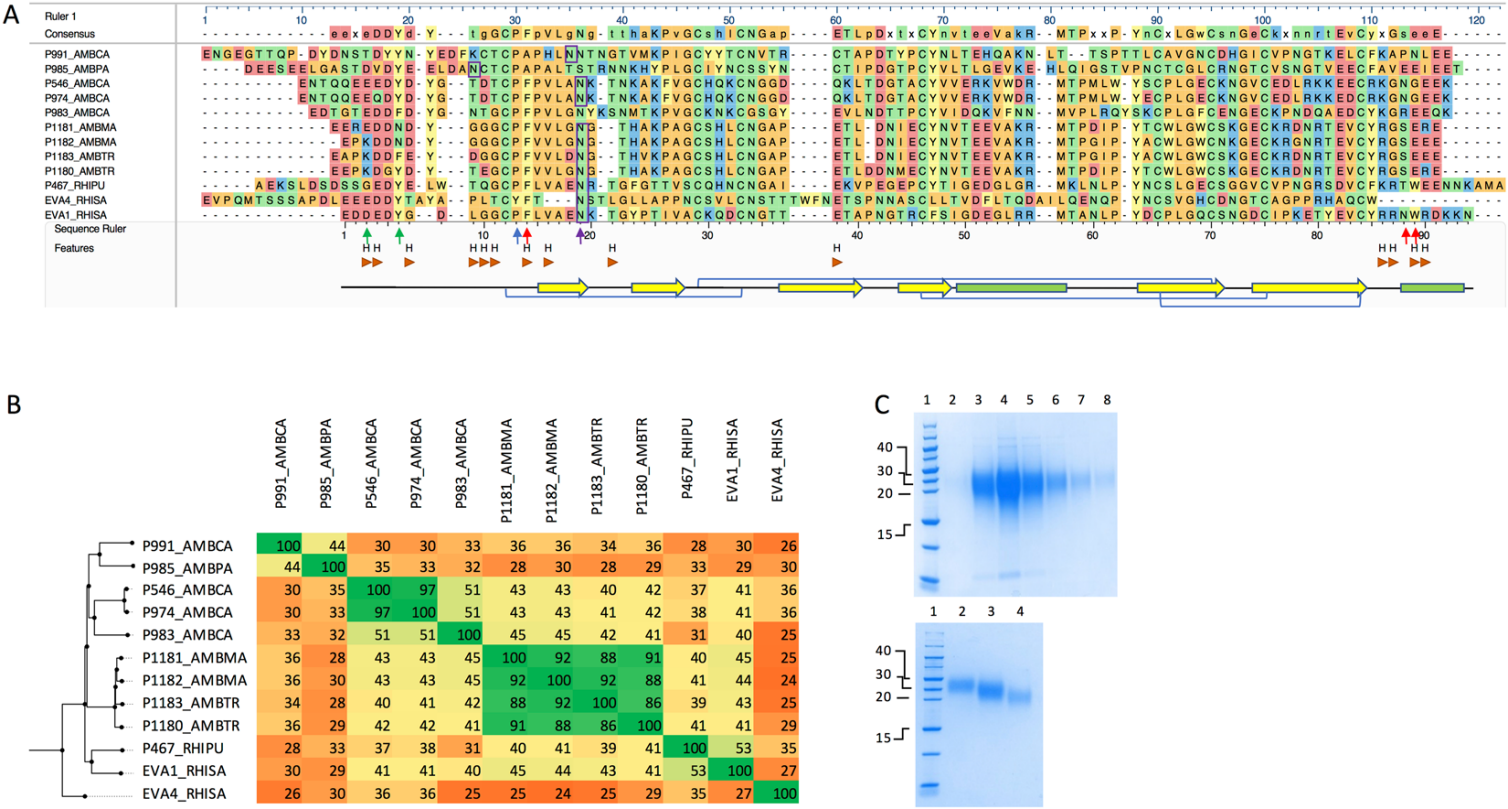
Analysis of CC-chemokine binding evasin sequences. (**A**) CLUSTAL-W alignment of CC-chemokine binding evasins with evasins 1 and 4 (EVA1, EVA4). Peptide sequence prefix indicates the identity, and suffix indicate the tick species as follows: RHISA and RHIPU – *Rhipicephalus sanguineus* and *pulchellus* respectively, and AMBPA, AMBCA, AMBMA, AMBTR - *Amblyomma parvum, cajennense, maculatum, triste*) respectively. Amino acid residues are color coded by physicochemical properties, and consensus sequence presented above the alignment. Evasin 1 H-bonds to CCL3 are shown as “H”. Evasin 1 structural motifs are indicated as yellow arrows (beta-sheet), green bars (alpha-helix), and blue connectors (disulfide bonds), and were taken from the analysis of the evasin 1:CCL3 structure 3FPU provided in PDBSum^18^. The blue arrow indicates the P13 residue in evasin 1 that targets the disulfide bond in CCL3^13^. Red arrows indicate F14, N88 and W89 residues in evasin 1 that make contact with CCL3. Green arrows indicate E16 and Y19 residues of evasin 4. The purple arrow indicates N19 in evasin 1, which is glycosylated. The conserved glycosylation sites at the N-terminus predicted using NetNGlyc are boxed in purple. (**B**) Sequence conservation between CC-chemokine binding evasins and EVA1 and EVA4 peptides. Percent identities between sequences are shown and were calculated by performing BLASTP with default parameters, and are color coded with green indicating high sequence conservation, yellow indicating medium, and orange indicating low conservation. CC-chemokine binding evasins are arranged according to the sequence-similarity based phylogenetic tree shown on the left of the figure. (**C**) Expression and purification of P991_AMBCA. Colloidal Coomassie stained gels of proteins fractionated by SDS-PAGE. Top panel: Elutions from nickel affinity column, lanes 3–8. Molecular weight ladder (kDa) lane 1. His-tagged P991_AMBCA has a predicted MW including tag of 15.7 kDa. Bottom panel: Fractions collected from size exclusion column chromatography of the pooled material obtained from the nickel affinity column.

Peptide lengths varied between 89 to 108 residues, molecular weights between 9.7 to 12 kDa, and pI between 4–5.2 (Table 1). Glycosylation site prediction indicates that all 10 evasins have between one to seven predicted N-glycosylation sites, and only three evasins have one or more predicted O-glycosylation sites (Table 1). Sequence alignment with CC-chemokine binding evasins 1 and 4 showed that all novel CC-chemokine binding evasins retained the Cys residues predicted to form disulfide bonds in evasin 1, and the Pro13 residue in evasin 1 that targets the disulfide bond in CCL3 (Fig. 3A). The arrangement of Cys residues is C-x(14,17) -C-x(3)-C-x(11,16)-C-x(17,20)-C-x(4)-C-x(4)-C-x(8)-C, with numbers in parentheses indicating spacing between Cys residues. Other residues in the novel evasins were poorly conserved with evasins 1 and 4, with overall identity ranging from 24 to 53% (Fig. 3B). Three residues in evasin 1 (F14, N88 and W89) are important for binding CCL3^19^. While F14 is conserved in 10 of 12 sequences, N88 and W89 are poorly conserved. Residues E16 and Y19 in evasin 4 are important for CCL3 binding. While Y19 is conserved (eight of 12 sequences), E16 is poorly conserved. The glycosylated residue in evasin 1 (N19) is highly conserved in nine of the other evasin sequences, which are also predicted to be glycosylated. A group of novel evasins, P1180 – P1183 show very high sequence identity (86–92%), and cluster together on a phylogenetic tree constructed from protein sequence similarity. P546 and P974 have 97% sequence identity, and also cluster together.

### Expression and purification of novel evasins

To further characterise the novel evasins biochemically and functionally, we expressed them as secreted proteins in mammalian cells fused to a StrepII:His tag. We used mammalian cells as these have previously been used to produce biologically active evasins suitable for use *in vitro* and *in* *vivo* ^30^. We used a plasmid vector designed for secretion^31^, and isolated expressed proteins from the tissue culture supernatant by nickel affinity followed by size exclusion chromatography (see Supplementary Fig. S2) to remove contaminating higher and lower molecular weight proteins observed after nickel affinity purification (see Fig. 3C top panel). Analysis of column chromatography fractions indicated that each evasin typically eluted in several fractions depending on molecular weight, and migrated at a larger molecular weight than expected (Fig. 3C, bottom panel), even accounting for the molecular weight of the affinity tag and linker, which was 3.4 kDa. This is consistent with the glycosylation predicted above, and is consistent with that observed with evasins 1 and 4 expressed in mammalian cells^18, 30^.

### Binding of novel evasins to human chemokines *in vitro*

**Figure 4:**
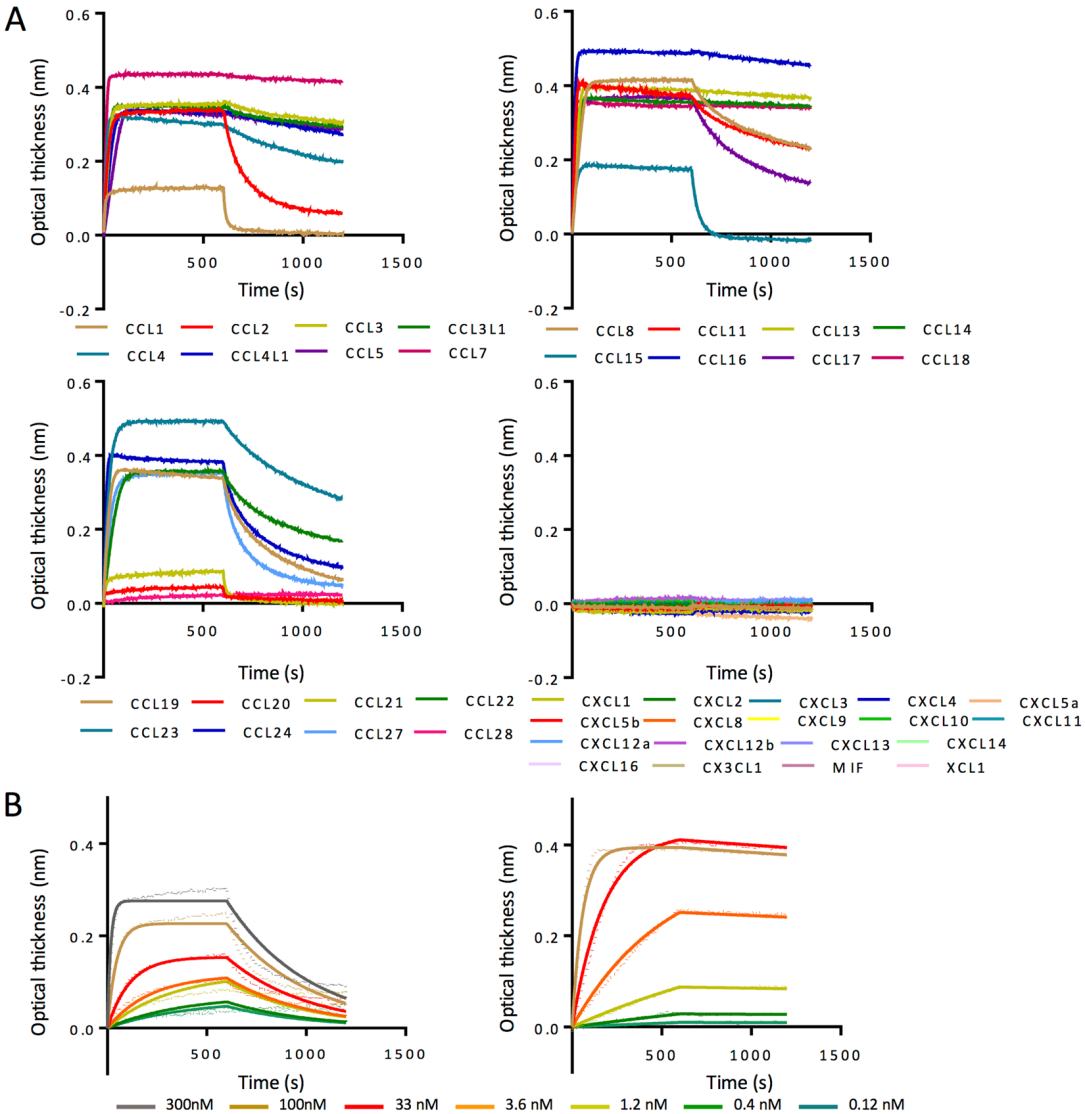
Characterization of P991_AMBCA by biolayer interferometry. (**A)** P991_AMBCA cross binding. Biolayer interferometry sensorgrams showing P991_AMBCA binding to different chemokines at 300 nM. Plots display optical thickness (y-axis, nm) versus time (x-axis, seconds). (**B**) P991_AMBCA binding to indicated chemokines. Biolayer interferometry sensorgrams showing P991_AMBCA binding to different doses (ranging from 300 nM to 0.4 nM) of chemokines CCL2 (left panel), and CCL13 (right panel). Plots display optical thickness (y-axis, nm) versus time (x-axis, seconds).

**Figure 5:**
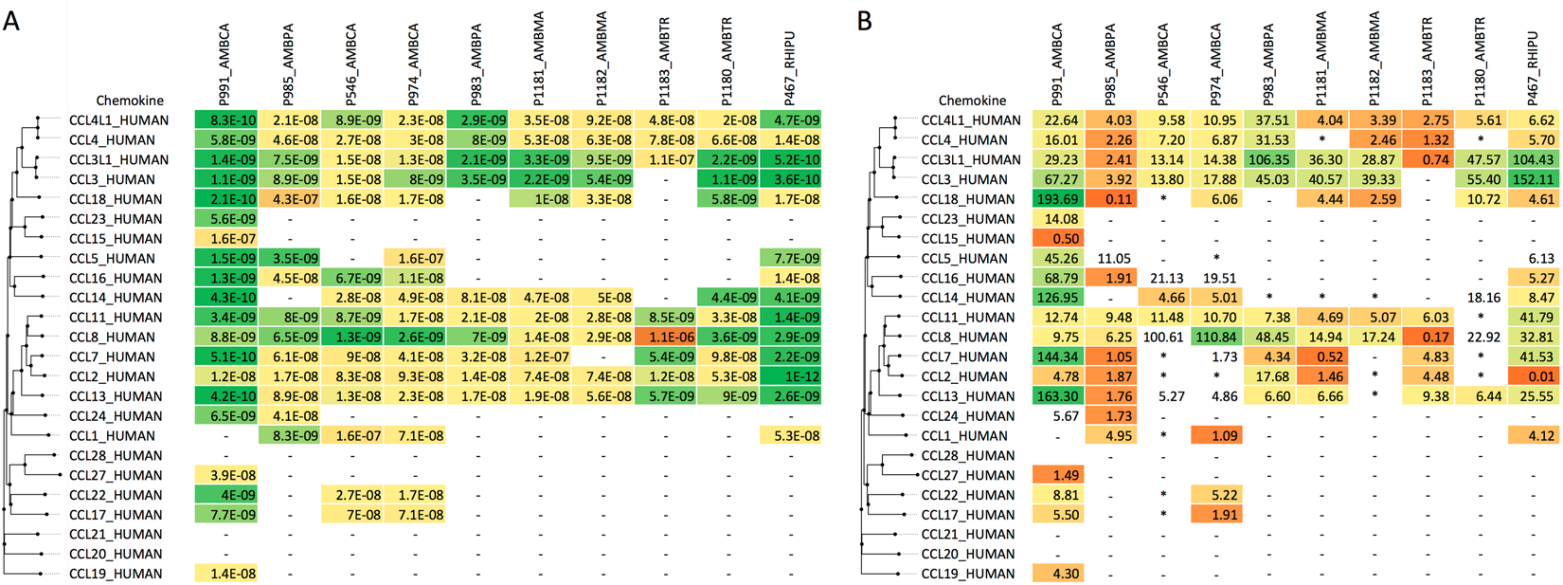
Binding of novel evasins to human CC chemokines. (**A**) Binding affinities (*K*_d_, moles/litre) of novel evasins to human CC-chemokines using biolayer interferometry. Higher affinity binding is indicated as shades of green, medium affinity as yellow, and lower affinity as shades of orange. Evasins and chemokines are arranged by sequence-similarity based phylogeny. - Indicates that binding was not detected at 300 nM chemokine concentration. (**B**) Dissociative half-life times (t_1/2_, minutes) of novel evasins with human CC-chemokines, calculated from biolayer interferometry off-rates (t_1/2_ = 0.693/(*k*_off_ × 60)^60^. - Indicates that binding was not detected at 300 nM chemokine concentration. *Indicates that *k*_off_ data were not available (as a steady-state fit was performed, see methods) and the dissociative half-life time could not be calculated. Evasins and chemokines are arranged by sequence-similarity based phylogeny. Longer half-lives are indicated as shades of green, medium as yellow, and shorter half-lives as shades of orange.

We assayed the binding of all 10 evasins using biolayer interferometry^32^ against a panel of human chemokines. In these assays evasins were bound to the probe through the C-terminal His tag. An initial screening assay was performed at 300 nM chemokine concentration to identify chemokines that bound to the evasin (Fig. 4A, and see Supplementary Table S1). While all novel evasins bound to CC chemokines as expected from the yeast-display results, none of the 10 evasins bound CXC, CX3 C or XC chemokines at a chemokine concentration of 300 nM. For determining binding affinities, we used serial dilutions of chemokines that bound respective evasins in the cross-binding screen (Fig. 4B). Binding affinities (*K*_d_) for all 10 CC-chemokine binding evasins against 24 CC-chemokines are shown in Fig. 5A. The number of chemokines bound, and the binding affinities vary significantly depending on the evasin. The binding profiles of novel evasins cannot be predicted by sequence similarity, with the exception of the very closely related group consisting of P1180, P1181, P1182 and P1183. A unifying feature that distinguishes these evasins from the binding profile reported for evasins 1 and 4 is their ability to bind CCL2 and CCL13. Importantly we observed a wide variation in chemokine off rates as seen in Fig. 4B. This variation in off rate translated into large variations in the dissociative half-life (Fig. 5B) that are not predicted by sequence similarity. For instance, for P991_AMBCA, the dissociation half-lives for the closely related chemokines CCL2, CCL13 and CCL7 were 4.78, 163 and 144 minutes respectively.

### Binding of P991_AMBCA to mouse chemokines *in vitro*

**Figure 6:**
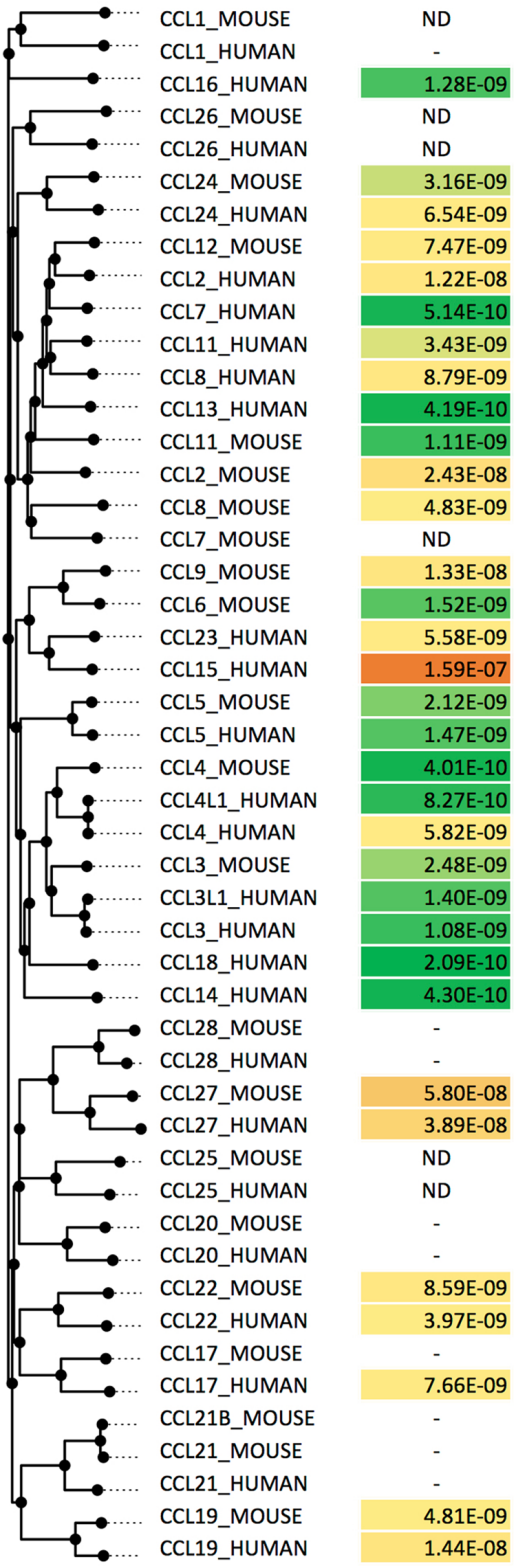
Binding of P991_AMBCA to mouse CC chemokines. Binding affinities (*K*_d_, moles/litre) of P991_AMBCA to mouse CC-chemokines using biolayer interferometry. ND indicates not done. - Indicates that binding was not detected at 300 nM chemokine concentration. Human and mouse CC-chemokines are arranged by sequence-similarity based phylogeny. Human chemokine binding to P991_AMBCA is as shown in Fig. 5, and shown here for comparison to mouse binding data. Higher affinity binding is indicated as shades of green, medium affinity as yellow, and lower affinity as shades of orange.

Our studies suggested that P991_AMBCA appears to be the evasin most suitable for development as a broad-spectrum CC-chemokine inhibitor (Fig. 5A), and we therefore characterized it in further detail. A significant consideration in the development of tool compounds and therapeutics is their ability to target not only human proteins but also those in animal models. Mouse – the most commonly used model for inflammatory human disease - has diverged from humans ~75 million years in the past and has a significantly divergent set of chemokines^33^. We therefore screened the novel evasins for binding to a panel of mouse CC-chemokines at 300 nM (see Supplementary Table S2) and found that several chemokines bind. We therefore characterized the binding of P991_AMBCA to mouse CC-chemokines using biolayer interferometry as described above (Fig. 6). Arranging P991_AMBCA binding data to a protein sequence-similarity based phylogenetic tree of mouse and human CC-chemokines shows that there is excellent correlation of evasin binding between mouse and related human chemokines. Thus P991_AMBCA appears to be suitable for mouse studies as a biological probe.

### Inhibition of chemokine function by P991_AMBCA

**Figure 7:**
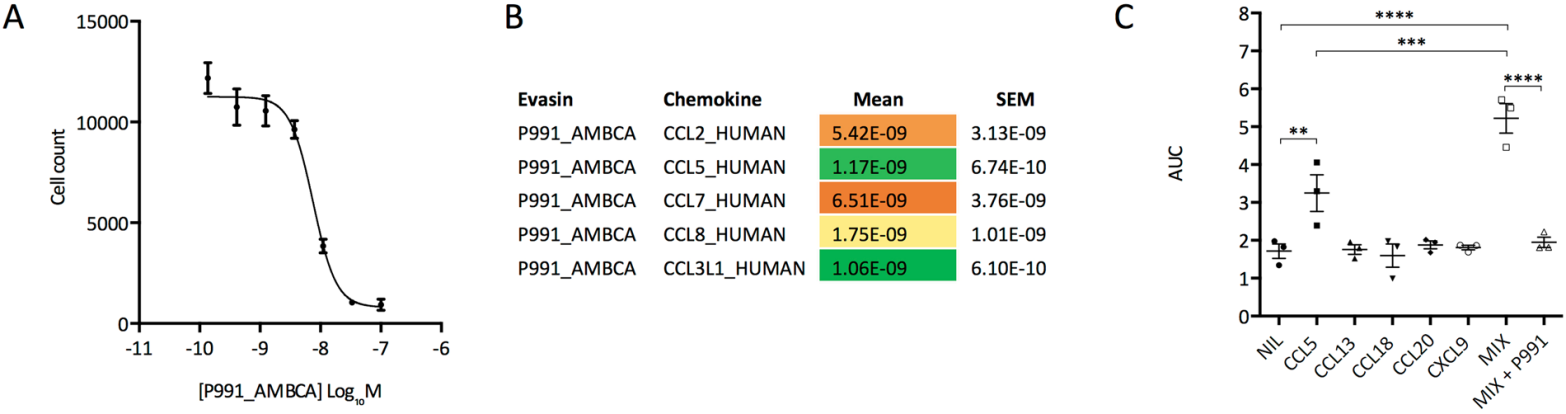
Functional neutralization of chemokine activity. (**A**) Neutralization of CCL2 induced THP-1 cell migration by P991_AMBCA. Y-axis shows cell count of THP-1 cells migrating through to the bottom chamber in response to EC_80_ dose of CCL2. Data (3 technical replicates) are shown as mean ± s.e.m. X-axis shows P991_AMBCA concentration (Log_10_ Molar). (**B**) Summary of P991_AMBCA IC_50_ data (moles/litre, mean, s.e.m. of 3 biological replicates analyzed as above) for chemokines that induce THP-1 cell migration. Lower IC_50_ is indicated as shades of green, medium as yellow, and high as shades of orange. (**C**) Neutralization of a complex chemokine mixture by P991_AMBCA. Y-axis shows AUC (area under progress curve) in response to indicated treatments shown on the x-axis. NIL indicates baseline migration without any chemokine. Results (mean ± s.e.m., and individual data points) of 3 independent biological replicate experiments are shown, and were analyzed with one-way ANOVA with Sidak’s multiple comparisons test and P values adjusted for multiple comparisons. *Indicates P < 0.05, **Indicates P < 0.01, ***P < 0.001, ****P < 0.0001.

We next determined if P991_AMBCA would inhibit chemokine function. For this we monitored its effects on the migration of THP-1 human monocyte cells in response to human chemokines in 96 well Boyden chamber assays. In initial experiments, we determined the EC_80_ of several chemokines, and found that these cells responded to low nM doses of CCL2, CCL3, CCL3L1, CCL5, CCL7 and CCL8 (see Supplementary Table S3). We next determined the effect of titrating in progressively increasing doses of evasins to determine IC_50_ (Fig. 7A,B). These results showed that P991_AMBCA inhibits the chemokine-induced migration of THP-1 cells with IC_50_ in the low nM range. Since migration under these conditions does not depend on GAG binding, it suggests that P991_AMBCA directly inhibits binding of chemokine to their receptors on THP-1 cells.

### Inhibition of a complex chemokine mixture by P991_AMBCA

The expression of multiple chemokines in disease tissue and chemokine receptor synergism is a source of network robustness. The orphan disease giant cell myocarditis is characterized by the expression of chemokines CCL5, CCL13, CCL18, CCL20 and CXCL9^34, 35^, and pathologically by the infiltration of the heart with monocytes and macrophages^36, 37^. Of these, CCL5^38^, CCL13^39^ and CCL20^40^ are known monocyte chemoattractants, CCL18 is an agonist of the receptor CCR8^41^ which is expressed on monocytes and macrophages^42^, and CXC and CC chemokines are known to synergize in monocyte chemotaxis^43^, suggesting that all five chemokines could play a role in disease pathogenesis. We found using a THP-1 monocyte cell-migration assay that in low doses (20 nM), CCL5 caused a small degree of THP-1 cell migration in comparison to baseline (NIL, absence of chemokine), but that the other chemokines individually did not (Fig. 7C). When added in combination these five chemokines caused a substantial increase in THP-1 migration over baseline, indicating a synergistic effect. We found that P991_AMBCA completely blocked the migration of THP-1 cells produced by the chemokine combination. These results indicated that P991_AMBCA, which targets three of the five chemokines (CCL5, CCL13, CCL18), is capable of overcoming the combinatorial and synergistic effects of these chemokines on THP-1 monocyte migration.

## Discussion

Mitochondrial phylogenetic studies indicate that ticks arose ~300 milion years (myr) ago and likely parasitized amphibians, and later reptiles, birds and mammals^44^. Metastriate hard ticks such as *Rhiphicephalus* and *Amblyomma* first appear in the late Jurassic (~134 myr), concomitant with the appearance of placental mammals^44^. Bioinformatic analyses of transcriptome data indicate that Metastriate ticks have evolved divergent evasin-like molecules^23, 24^. Such novel peptides could be valuable tool compounds and therapeutics.

We developed a peptide display approach to rapidly select chemokine binders from putative evasins identified in tick salivary transcriptomes. Peptide display technologies have in common the ability to physically link peptide sequences with the encoding DNA, and are powerful ways to select a variety of molecular shapes based on protein interactions. Approaches that have been used include yeast surface display – used to select antibody fragments^25^, monobodies^45^, knottins^46^, tick salivary antigens^47^, and tick gut peptides interacting with *B. burgdorferi* proteins^48^, mammalian cell surface display used for single chain antibodies^49^, bacterial surface display used for nanobodies^50^, cyclotides^51^ and anticalins^52^, phage display used for antibodies^53^, monobodies^54^ and evasins^19^, and mRNA display – used for cyclic peptides^55^. A major advantage of yeast surface display for eukaryotic secreted proteins is that unlike mammalian cell surface display, large stably transfected yeast libraries can easily be made, and as protein disulfide isomerases and chaperones are present in the secretory pathway of yeast^25^, they do not need to be artificially introduced as in phage or bacterial display^19^. We therefore explored if evasins could be rapidly identified and functionally characterised using yeast surface display.

Our initial studies showed that evasins of two unrelated structural classes can be displayed on yeast surface in either N- or C-terminally tagged orientations. The display orientation affected the ability of the evasin to recognize the target chemokine, confirming previous studies^56^. These experiments provided proof-of-concept that surface displayed evasins can be identified using flow sorting with a fluorescent chemokine probe. We created a library consisting of 24 members, screened it with the chemokine CCL5, as it is important in the pathogenesis of cardiovascular disease, and successfully recovered novel evasins. These yeast display studies confirmed that the nature and location of the surface display tag affected the chemokine-binding function of an evasin unpredictably, and support the use of display tags in different orientations when constructing such libraries. Moreover, they mean that a clone need not be recovered in either orientation in a screen as the display tag may interfere with binding.

Comparison of the chemokine binding pattern of C-terminally tagged P467_RHIPU in yeast with that of C-terminally tagged purified protein showed that they were similar, with both showing binding to CCL1, CCL2, CCL3, CCL5, CCL8, CCL11 and CCL18. However, there were some discrepancies, with the purified protein not binding CCL17, CCL19 and CCL22. We do not understand the mechanisms of the discrepancy, but they possibly arise from underlying differences in the assays and in the C-terminal tag used. Our unpublished data (J.R.O.E., A.K., S.B.) also indicate that altering the position of the purification tag in mammalian expressed proteins affects binding specificity, and indeed this phenomenon could potentially be used to engineer the desired chemokine binding properties of an evasin. By extending our screening studies with other chemokines, and our library to >350 members, we successfully identified a total of 26 CC-chemokine binding evasins of which we have characterised 10 in detail here. These studies suggest that many putative evasins identified by bioinformatics may not have high enough affinity for chemokines used in the screens performed to date in order for them to be selected by yeast surface display, but may bind other chemokines or have functions other than chemokine binding.

Analysis of the novel CC-chemokine binding evasins showed that they resemble evasin 1 in retaining Cys residues that are necessary for intra-chain disulfide bond formation, and conserve the Pro residue that targets the invariant disulfide in chemokines. The novel evasins are however remarkably dissimilar to evasin 1 or 4 in primary structure. Conservation of the Cys residues suggests that these CC-chemokine binding evasins have a common disulfide bonded structure that arose in a common ancestor. The lack of conservation of other key residues suggest that these novel evasins evolved different pharmacophores to bind target chemokines.

Like evasins 1 and 4, the novel evasins have multiple predicted glycosylation sites^18, 30^, and consistent with this,  migrate at a significantly higher apparent molecular weight. The striking conservation of the N-terminal glycosylation site corresponding to N19 in evasin 1 suggests that there is evolutionary pressure maintaining glycosylation at this site. As glycosylation is not essential for chemokine recognition by an evasin^18^, a possible explanation is that such glycosylation reduces access to neutralizing antibodies by creating a “glycan-shield”^57, 58^, and likely explains the lack of detectable antibody response following evasin therapy^59^.

Binding assays performed using purified proteins showed that the novel evasins bound to a range of CC chemokines with high affinity. Remarkably, the off-rates and consequently the dissociative half-lives of the interactions showed wide variations even with closely related chemokines and closely related evasins. The large dissociative half-lives, in excess of a 100 minutes observed in 5 of 10 evasins, with *k*_off_ similar to that of antibody-antigen interactions, suggests that for these interactions there is a change in conformation leading to greater steric complementarity between evasin and chemokine after binding, and resulting in a very slowly dissociating complex. Indeed, conformational changes in both partners has been observed in the interaction between evasin 1 and CCL3^18^. Advantages of a long residence time for a pharmacological agent include prolonged action, complete physiological inhibition requiring endogenous synthesis of target for recovery, and reduced off-target effects^60, 61^. Notably, chemokine interaction with cell surface glycosaminoglycans (GAGs) is known to promote chemokine stability, is essential for chemokine function *in vivo* ^62^, and can result in conformational changes in chemokine structure and oligomerization^63^. While the ability of an evasin to bind GAG-bound chemokines is not known, it is conceivable that the prolonged residence time of certain evasins may be an evolutionary adaptation to neutralizing GAG-bound chemokines.

The remarkable differences in dissociative half-lives between similar chemokines (e.g. the binding of P991_AMBCA to CCL2 and CCL13), suggest that different evasin-chemokine binding mechanisms must exist. For instance, the ability to block eosinophil chemotaxis by CCL13 could be very relevant to the role that eosinophils play in parasitic infestation^64^. This is also suggested by the different combinations of chemokines that are bound by individual evasins, and also by the ability of certain evasins to target a very large number of structurally distinct chemokines. These mechanisms can be understood by structural analyses of chemokine – evasin complexes, and we have initiated such studies. Our studies, and those previously reported with evasins 1 and 4, also indicate that binding by an evasin predicts its ability to neutralize the chemotactic function of a target chemokine. In the case for evasins 1 and 4, binding occurs to the N-terminus of CCL3^18, 19^, which prevents access to the CRS2 domain of the receptor. It is possible that the novel evasins reported here also function in a similar manner, and needs further exploration.

A key novel feature of this group of evasins, absent in evasins 1 and 4, is their ability to bind and neutralize CCL2 and CCL13, in addition to other CC-chemokines. Further structural studies are needed to understand the mechanism used in targeting these two chemokines that makes them distinct from evasins 1 and 4. The ability to target CCL2 - a monocyte recruiting chemokine expressed in the tumour microenvironment^65^, myocarditis^66^, myocardial infarction^67^ and stroke^68^ - in addition to other chemokines makes these evasins potentially useful in disrupting the chemokine network in these diseases. Similarly, CCL13 is expressed in myocarditis^35^, myocardial infarction^69^ and idiopathic lung fibrosis^70^, making these new evasins useful tool compounds and potential therapeutics in these and other chemokine-driven inflammatory disease. Our data also indicate that certain evasins such as P991_AMBCA can bind related chemokines from species such as mouse and human that diverged ~75 million years ago. This is not unsurprising as ticks such as *Amblyomma cajennense* parasitize not only humans, but also dogs, cattle, birds, and capybaras^24^. The ability to bind related targets across different species is relevant in the development of new therapeutics in pre-clinical animal models that then have to be translated into human disease.

Key features of the chemokine network that have evolved to make it robust to attack by pathogens but frustrate the development of novel therapeutics are the multiplicity of chemokines typically expressed in disease, the polyvalent nature of chemokine - receptor interactions, and expression of several chemokine receptors on a single cell type that can act synergistically. Our studies using a small group of five chemokines that are expressed in giant cell myocarditis show that they can indeed act synergistically on the recruitment of monocytes, and also that a single evasin that targets three of these five chemokines can abolish monocyte recruitment by the chemokine combination. Future studies to determine the *in vivo* concentration of an evasin necessary to neutralize myocarditis chemokines are necessary to extend our *in vitro* data.

In conclusion, we have developed yeast surface display as an approach to rapidly identify proteins from tick saliva that bind and neutralize members of the mammalian chemokine network. These proteins could be developed as potential therapeutics to target diverse inflammatory diseases that are driven by chemokines. More broadly, the explosion of transcriptome sequence information from diverse biological resources requires efficient proteomic approaches for identification and functional characterisation of inter-species protein interactions. Our studies provide evidence that yeast surface display could be used to systematically identify such protein interactions, and rapidly mine potential therapeutics from organisms that have, as evolutionary adaptations, developed peptide arsenals in “endless forms most beautiful”^71^.

## Methods

### Bioinformatics

Tick transcriptome datasets were accessed at NCBI (http://blast.ncbi.nlm.nih.gov/, non-redundant and transcriptome shotgun assembly databases), and searched using three iterations of psiBLAST^72^, using the mature peptide sequences of evasins 1 and 4, in order to detect biologically relevant but weak protein similarities. Peptides with e-value < 1E-5, possessing a signal peptide defined by SignalP 3.0^73^, and mature peptide length < 200 amino acid residues were classed as putative evasins.

### Plasmids

Yeast expression shuttle plasmids and plasmid libraries were assembled from constituent idempotent parts (promoter, signal peptide, cDNA, surface display tags, epitope tags, terminator, replication origins, antibiotic and/or auxotrophic selection markers) that we have developed as part of a synthetic biology toolkit for use in GoldenGate cloning^74^. Constituent parts were generated using PCR or gene synthesis (GeneWiz, South Plainfield, NJ, USA), cloned into plasmids, and sequenced to confirm lack of mutations. Evasin part overhangs were designed so that they were compatible with either N- or C-terminal surface display tags. Plasmid assembly was performed using the single pot GoldenGate/GoldenBraid cloning method^74^ that we modified to incorporate a negative selection *ccdb* gene cassette (Invitrogen) for improved cloning efficiency, enabling library construction. Yeast expression shuttle plasmids contained a yeast GAL1 promoter, synthetic AMYG_RHIOR signal peptide, 2-micron replication origin and ADH1 terminator, and TRP auxotrophic marker. Surface display tags were AGA2_YEAST (from pCTCon2^25^) or SAG1_YEAST (residues 332–650^27^), and were separated from the evasin cDNA using a linker encoding (GGGGS)x3. Yeast shuttle plasmids and libraries were created in three configurations, with N-terminal AGA2_YEAST, C-terminal AGA2_YEAST, and C-terminal SAG1_YEAST surface display tags. Each plasmid library was made to achieve >10-fold overrepresentation of the evasin library inserts. Mammalian expression plasmids were created in the vector pHLSec^31^ (kind gift from Radu Aricescu) by Infusion cloning (Clontech) following the manufacturers recommendations. Evasin expression plasmids contained a C-terminal G4S linker and strep II-8xHis protein purification tag (GGGGGSGGGGSGGASAWSHPQFEKLEHHHHHHHH)^75^ and N-terminal ETG sequence from plasmid pHLSec.

### Yeast surface display

Yeast surface display was performed essentially as described^28^. EBY100 yeast (Invitrogen) were transformed with shuttle plasmids. For library screens, we achieved >10 fold overrepresentation of each unique evasin. Yeast cells were induced with galactose, 5E5 cells labelled in 1 ml PBS with biotinylated chemokines (Almac, 20 uM, 2.75 μl) and streptavidin-Alexa647 (2mg/ml, Invitrogen, 1.25 μl), incubated for 30 minutes on ice in the dark, spun at 8000 rpm for 5 minutes in a benchtop microcentrifuge, the pellet resuspended in 200 μl PBS, and sorted using a MoFlo FACS system with a gate defined by a negative control that omitted the chemokine. Cells recovered were re-grown and sorted a second time. For initial experiments, shuttle plasmids were isolated from individual sorted yeast colonies using a DNA prep kit (The Epigenetics Company), rescued into electrocompetent DH5α cells by electroporation, plasmid DNA miniprepped, and evasin insert identified by capillary sequencing followed by BLAST searching of a local database. Subsequently, we adopted a more efficient method wherein we directly amplified the insert using PCR typically from 24 independent colonies, and sequenced them prior to plasmid rescue. Rescued plasmids were re-transformed to yeast to confirm chemokine binding by FACS analyses as above but using a 96-well ATTUNE NXT system (Life Technologies). FACS data was analysed using FlowJo v10.02.

### Evasin peptide sequence analysis

Peptide sequence alignments were performed using Clustal-W in Megalign Pro (DNAStar version 12.3.1, DNAStar Inc.), and a sequence-similarity based phylogenetic tree exported to FigTree (version 1.4.2, http://tree.bio.ed.ac.uk/software/figtree/). Glycosylation site prediction was performed using NetNGlyc1.0 (http://www.cbs.dtu.dk/services/NetNGlyc/) and NetOGlyc4.0 (http://www.cbs.dtu.dk/services/NetOGlyc/)^76^. Peptide molecular weight and isoelectric point (pI) were calculated at ExPASy (http://web.expasy.org/compute_pi/). Chemokine sequence alignments and sequence-similarity based phylogenetic trees were constructed using MUSCLE in Megalign Pro.

### Cell lines

HEK293F cells were a gift from Nicola Burgess-Brown (University of Oxford), and THP-1 cells were from Sigma. Cell lines were confirmed mycoplasma free by a kit (MycoAlert™, Lonza) and by DAPI staining and authenticated functionally by protein production for HEK293F, and chemokine – induced migration for THP-1.

### Evasin protein production

HEK293F cells were transiently transfected using polyethylineimine (Sigma) and cultured in Freestyle™ 293 expression medium (ThermoFisher) at 37 °C, 8% CO_2_, with shaking for 5 days. Proteins were purified from filtered supernatants by gravity flow through Nickel charged IMAC Sepharose 6 Fast Flow resin (GE Healthcare), equilibrated with binding buffer (20 mM NaPO4, 500 mM NaCl, pH 7.4), washed after binding with wash buffer (20 mM NaPO4, 1 M NaCl, 20 mM imidazole, pH 7.4) and eluted in elution buffer (20 mM NaPO4, 500 mM NaCl, 500 mM imidazole, pH 7.4). Elutions were concentrated using an Amicon Ultra-15 Centrifugal Filter Unit with Ultracel-3 membrane (Millipore), and purified by size exclusion chromatography on an AKTA Start system using HiLoad 16/600 Superdex 75 (GE Healthcare), in SEC buffer (phosphate buffer saline (Sigma), with 150mM NaCl). Fractions showing absorption at 280 nm were analyzed by electrophoresis on a 12% SDS-PAGE gel, and stained with colloidal Coomassie.

### Biolayer interferometry cross-binding screen

Biolayer interferometry was performed on an OctetRed® system at 30 °C using the dip and read Ni-NTA biosensors (ForteBio). The assay buffer used was 10 mM Na_2_HPO_4_, 1.8 mM KH_2_PO_4_, 2.7 mM KCl, 500 mM NaCl, 0.1% BSA, 0.002% TWEEN-20, pH 7.4. Assay buffer was used to establish the response baseline (180 seconds). The temperature condition was recommended by the manufacturer and buffers optimized to minimize non-specific binding of chemokines to the sensor (J.R.O.E., A.K. & S.B., unpublished data). Purified His-tagged evasin was immobilized on the Ni-NTA sensor using 1 μM evasin in assay buffer (500 seconds). After washing the sensor with assay buffer (60 seconds), the sensor was then subjected to 300 nM chemokine in assay buffer (600 seconds) to record the association phase. The cross-binding screen was performed as a single experiment against all human chemokines with the exception of CCL25, CCL26, and CXCL16, where the chemokine non-specifically bound to the sensor, and CXCL17, CXCL4L1, and XCL2, which were not available from Peprotech. Assay buffer was then reapplied (600 seconds) to measure the dissociation. The sensor was then exposed to 10 mM glycine, pH 1.7 for five seconds and assay buffer for five seconds three times to strip the sensors of nickel and protein. The sensor was regenerated by placing the sensor into 10 mM NiCl_2_ for 60 seconds and then reused. Reference experiments were performed by placing a sensor with evasin alone loaded into buffer to account for baseline drift which was then subtracted. Data was processed in ForteBio Data Analysis 9 software. A similar protocol was used for mouse chemokines.

### Biolayer Interferometry *K*_d_ and *k*_off_ determination experiments

Measurements were carried as above. Each experiment was performed once, with a range of chemokine dilutions from 300 nM to 0.4 nM. Briefly, after washing the sensor with assay buffer for 60 seconds, the sensors were then subjected to a serial dilution of chemokine in assay buffer for 600 seconds to record the association phase. Assay buffer was then reapplied to measure the dissociation for 600 seconds. To account for non-specific binding to the sensor, a non-interacting reference protein of sequence DGGQRNAICRLPPDEGICRASIPRFYFNPAEGKCSFFIYGGCEGNENNFETIEECEKTCGEPERPSDFEGADFETGCAPKPQRGFCKGFLDHWFFNVTSGECEAFLYSGCGGNDNNYESKEECEIACKLTGGASAWSHPQFEKLEHHHHHHHH was loaded on to the sensor and any binding observed subtracted. This protein was shown to display no binding to any of the chemokines tested and was produced in house as above. Data was processed in ForteBio Data Analysis 9 software. Association (*k*_on_), dissociation (*k*_off_), and affinity (*K*_d_) constants were determined by using the 1:1 binding-model, and global fitting, followed by ‘R_max_ unlinked by sensor’ to allow independent fitting of R_max_ (maximal signal response upon saturating binding of the partner to the immobilized protein). For certain chemokine-evasin interactions where a curve fit was not possible, a steady state analysis was performed using ForteBio Data Analysis 9 software to obtain the *K*_d_, from the equilibrium response, and here *k*_off_ and *k*_on_ were not calculated. Data with poor curve fits (R^2^ < 0.9) were excluded. Dissociation half-life (minutes) was calculated as 0.693/ (*k*_off_ × 60) from the off-rate (*k*_off_, s^−1^) obtained from biolayer interferometry analysis^60^.

### Cell migration assays

Effective concentrations (EC) EC_80_ and EC_50_ for each chemokine was determined in three technical and three biological replicates essentially as described^77^, using a 96 well transwell migration plate (5 μM pore size, Corning). Chemokines (0–100 nM (Peprotech), in 150 μl RPMI-1640 + L-glu (2mM) + 0.5% heat treated fetal bovine serum, all from Sigma) were placed in the bottom chamber. Cells (THP-1, 3E5, in 50 μl RPMI-1640 + L-glu (2 mM) + 0.5% heat treated fetal bovine serum)) were placed in the top chamber, and incubated at 37 °C, 5% CO_2_ for 4 hours. The migration plate was shaken at 850 r.p.m. for 10 minutes, and media from bottom plate transferred to a V-bottomed 96 well plate. Cells were counted on a ATTUNE flow cytometer using a FSC versus SSC dot plot, and data analysed in GraphPad Prism fitting an agonist response curve with 4 parameters.

IC_50_ for each evasin was determined in three technical and three biological replicates essentially as described^77^ using the above system. Chemokine (EC_80_ dose) and evasin (0–100 nM doses) were added to the bottom chamber and incubated for 30 min at 37 °C before beginning the cell migration assay. Data (3 technical replicates for each IC_50_ determination) were analysed in GraphPad Prism fitting an inhibitor response curve with 4 parameters. The mean IC_50_ from 3 biological replicates was then calculated. For studies where we investigated neutralization of a complex chemokine mixture we used an Incucyte ZOOM® chemotaxis system and recorded progress curves over 24 hours following the manufacturer’s instructions, and calculated the area under the progress curve.

### Statistical analyses

Summary statistics were calculated in GraphPad Prism. Tests of significance were calculated using ordinary one-way ANOVA, assuming Gaussian distribution and equal variance, in GraphPad Prism, with Sidak’s multiple comparisons test (Fig. 7C). For each case the P value (probability of a type I error) reported was adjusted for multiple comparisons. The threshold (alpha) for a type I error was P < 0.05. For all cell based experiments sample sizes were n = 3 technical and n = 3 biological replicates, and were not powered to detect a pre-specified effect size. Investigators were not blinded to group allocation.

### Data availability

The authors declare that data supporting this manuscript are available either within the manuscript or as Supplementary Information.

## Electronic supplementary material

Supplementary Information
