# Author Correction: Yeast surface display identifies a family of evasins from ticks with novel polyvalent CC chemokine-binding activities

**DOI:** 10.1038/s41598-021-92868-8

**Published:** 2021-07-07

**Authors:** Kamayani Singh, Graham Davies, Yara Alenazi, James R. O. Eaton, Akane Kawamura, Shoumo Bhattacharya

**Affiliations:** 1 RDM Division of Cardiovascular Medicine and Wellcome Trust Centre for Human Genetics, University of Oxford, Roosevelt Drive, Oxford, OX3 7BN UK; 2 Dept of Chemistry, University of Oxford, Mansfield Road, Oxford, OX1 3TA UK

Correction to: *Scientific Reports* 10.1038/s41598-017-04378-1, published online 27 June 2017

The original version of this Article contained errors in Table 1, where a GenBank accession number was incorrect for Evasin ‘P1181_AMBMA’ and a GenBank accession number was missing for Evasin ‘P983_AMBCA’.

The correct and incorrect values appear below.

Incorrect:

**Table:** 

Evasin	Accession
P991_AMBCA	JAC19654.1
P985_AMBPA	JAC24842.1
P546_AMBCA	JAC18992.1
P974_AMBCA	JAC18993.1
P983_AMBCA	
P1181_AMBMA	JAC19634.1
P1182_AMBMA	AEO33551.1
P1183_AMBTR	JAC29608.1
P1180_AMBTR	JAC29349.1
P467_RHIPU	JAA60786.1

Correct:

**Table:** 

Evasin	Accession
P991_AMBCA	JAC19654.1
P985_AMBPA	JAC24842.1
P546_AMBCA	JAC18992.1
P974_AMBCA	JAC18993.1
P983_AMBCA	JAC19634.1
P1181_AMBMA	AEO33444.1
P1182_AMBMA	AEO33551.1
P1183_AMBTR	JAC29608.1
P1180_AMBTR	JAC29349.1
P467_RHIPU	JAA60786.1

The original Article has been corrected.

