# Supplementary material for: Yeast surface display identifies a family of evasins from ticks with novel polyvalent CC chemokine-binding activities
Source: Sci Rep. 2017 Jun 27;7:4267. doi: 10.1038/s41598-017-04378-1 (PMC5487423; doi:10.1038/s41598-017-04378-1)
Supplement: Supplementary file 1 — Supplementary Information [file 41598_2017_4378_MOESM1_ESM.doc]

**Yeast surface display identifies a family of evasins from ticks with novel polyvalent CC chemokine-binding activities**

Kamayani Singh^1,2^, Graham Davies^1,2^, Yara Alenazi^2^, James R.O. Eaton^2,3^, Akane Kawamura^2,3^ & Shoumo Bhattacharya^2*^.

^1^Joint first authors

*Corresponding author

^2^RDM Division of Cardiovascular Medicine and ^3^Department of Chemistry, Wellcome Trust Centre for Human Genetics, University of Oxford, Roosevelt Drive, Oxford OX3 7BN, United Kingdom

**Correspondence to:** Shoumo Bhattacharya (sbhattac@well.ox.ac.uk), Wellcome Trust Centre for Human Genetics, University of Oxford, Roosevelt Drive, Oxford OX3 7BN, UK

**
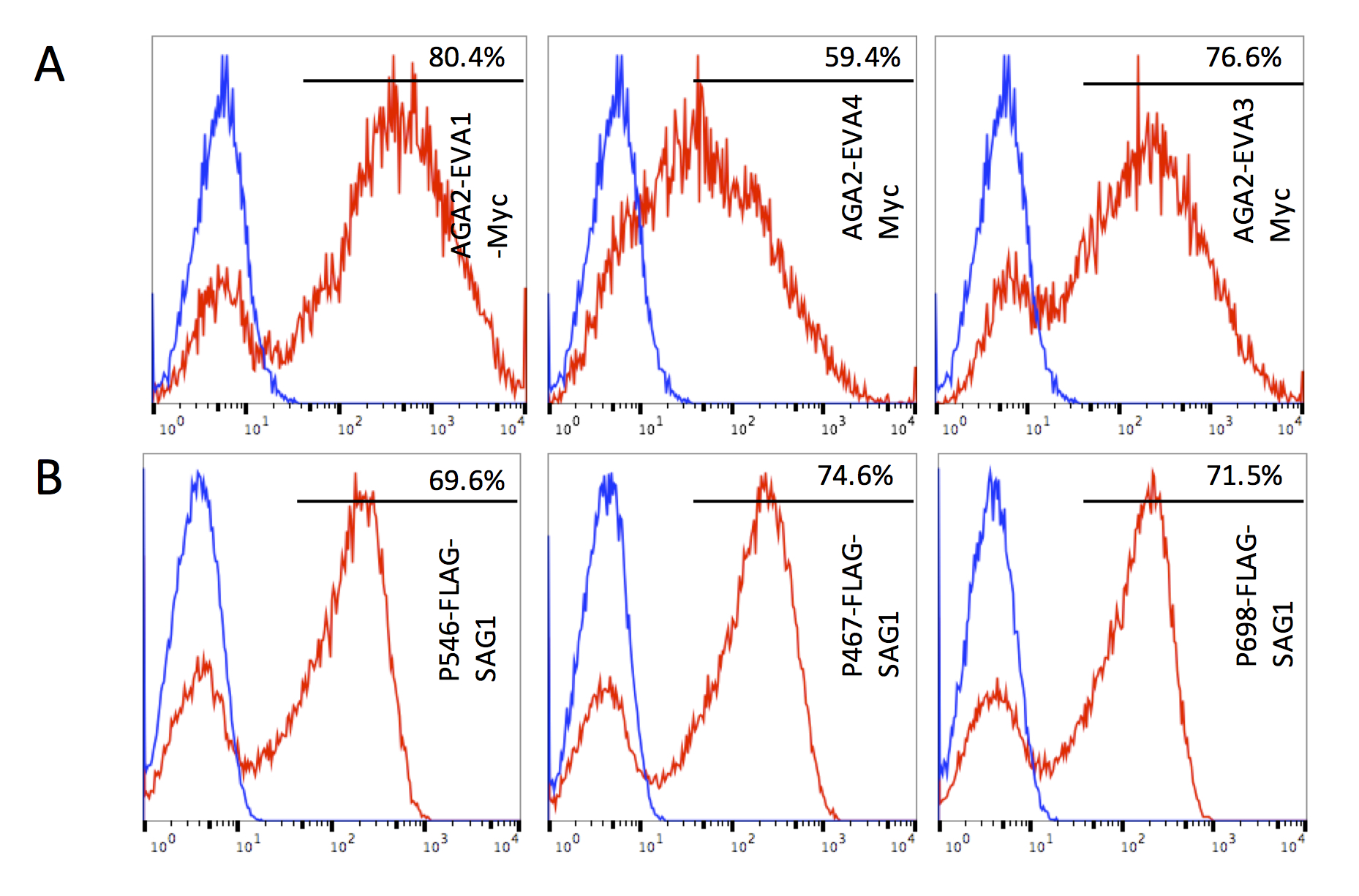
Supplement Figure S1. Fluorescent labelling of yeast using epitope tags.**

**A** Fluorescence profiles (red curves) of yeast displaying evasin 1 (left), evasin 4 (middle) and evasin 3 (right) panels incubated with anti-myc monoclonal antibody 9E10 (Roche), and then with goat-anti mouse polyclonal antibody conjugated with AF488 (Life Technologies, red curves). Surface display tags were placed at the evasin N-terminus (AGA2) with the myc epitope at the C-terminus. y-axis shows cell count (side scatter), and x-axis the fluorescence intensity on a log-scale. The blue curve is the profile of identical yeast treated with goat-anti mouse polyclonal antibody tagged with AF488 alone, and is used to determine background. The percentages of cells exceeding background are indicated in each panel.

**B** Fluorescence profiles (red curves) of yeast displaying P546 (left), P467 (middle) and P698 (right) panels incubated with monoclonal anti-FLAG M2--FITC (red curves). Surface display tags were placed at the evasin C-terminus (SAG1) with the FLAG epitope also at the C-terminus. y-axis shows cell count (side scatter), and x-axis the fluorescence intensity on a log-scale. The blue curve is the profile of identical yeast treated with mouse IgG1 FITC conjugate antibody as isotype control, and is used to determine background. The percentages of cells exceeding background are indicated in each panel.


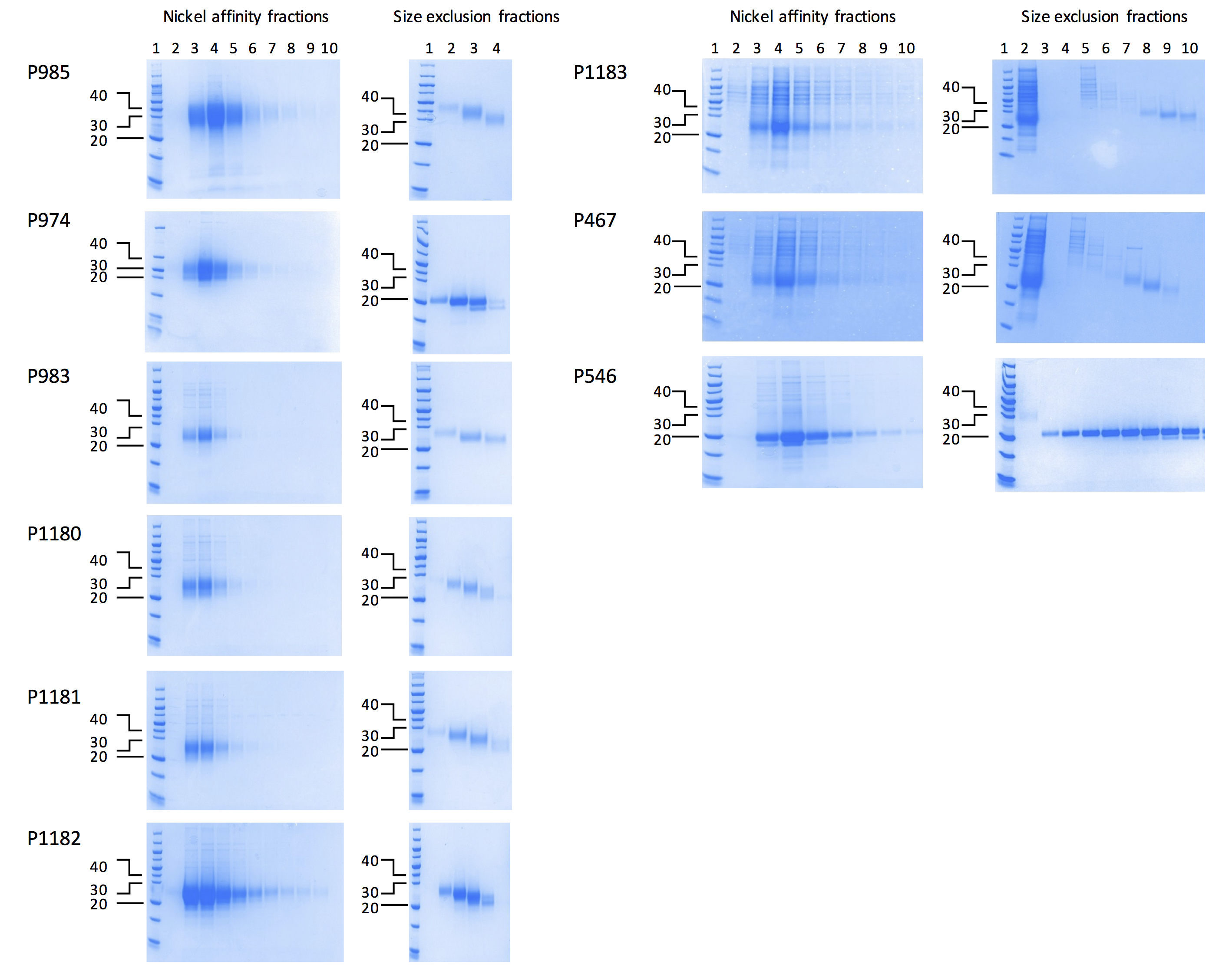


**Supplement Figure S2. Expression and purification of evasins.**

Colloidal Coomassie stained gels of individual evasin proteins fractionated by SDS-PAGE. Left panels: Elutions from nickel affinity column. Molecular weight ladder (kDa) lane 1. His-tagged proteins have a MW of between 20-30 kDa. Right panels: Fractions collected from size exclusion column chromatography of the pooled material obtained from the nickel affinity column.

Supplementary Tables

| **Supplementary Table S1: Binding of evasins with human chemokines at 300 nM using biolayer interferometry.** | | | | | | | | | | |
| --- | --- | --- | --- | --- | --- | --- | --- | --- | --- | --- |
| **Chemokine** | **P991_AMBCA** | **P985_AMBMA** | **P546_AMBCA** | **P974_AMBCA** | **P983_AMBPA** | **P1181_AMBMA** | **P1182_AMBMA** | **P1183_AMBTR** | **P1180_AMBTR** | **P467_RHIPU** |
| **CCL1_HUMAN** | Y | Y | Y | Y | Y | Y | Y | Y | Y | Y |
| **CCL2_HUMAN** | Y | Y | Y | Y | Y | Y | Y | Y | Y | Y |
| **CCL3_HUMAN** | Y | Y | Y | Y | Y | Y | Y | Y | Y | Y |
| **CCL4_HUMAN** | Y | Y | Y | Y | Y | Y | Y | Y | Y | Y |
| **CCL5_HUMAN** | Y | Y | Y | N | Y | N | N | Y | Y | Y |
| **CCL7_HUMAN** | Y | Y | Y | Y | Y | Y | Y | Y | Y | Y |
| **CCL8_HUMAN** | Y | Y | Y | Y | Y | Y | Y | Y | Y | Y |
| **CCL11_HUMAN** | Y | Y | Y | Y | Y | Y | Y | Y | Y | Y |
| **CCL13_HUMAN** | Y | Y | Y | Y | Y | Y | Y | Y | Y | Y |
| **CCL14_HUMAN** | Y | N | Y | Y | Y | Y | Y | N | Y | Y |
| **CCL15_HUMAN** | Y | N | N | N | N | N | N | N | N | N |
| **CCL16_HUMAN** | Y | Y | Y | Y | Y | N | N | N | N | Y |
| **CCL17_HUMAN** | Y | N | Y | Y | N | N | N | N | N | N |
| **CCL18_HUMAN** | Y | Y | Y | Y | Y | Y | Y | Y | Y | Y |
| **CCL19_HUMAN** | Y | N | N | N | N | N | N | N | N | N |
| **CCL20_HUMAN** | Y | N | N | N | N | N | N | N | N | N |
| **CCL21_HUMAN** | Y | N | N | N | N | N | N | N | N | N |
| **CCL22_HUMAN** | Y | N | Y | Y | N | N | N | N | N | N |
| **CCL23_HUMAN** | Y | N | N | N | Y | N | N | N | N | Y |
| **CCL24_HUMAN** | Y | Y | N | N | N | N | N | N | N | Y |
| **CCL25_HUMAN** | * | * | * | * | * | * | * | * | * | * |
| **CCL26_HUMAN** | * | * | * | * | * | * | * | * | * | * |
| **CCL27_HUMAN** | Y | N | N | N | N | N | N | N | N | N |
| **CCL28_HUMAN** | N | N | N | N | N | N | N | N | N | N |
| **CCL3L1_HUMAN** | Y | Y | Y | Y | Y | Y | N | Y | Y | N |
| **CCL4L1_HUMAN** | Y | Y | Y | Y | Y | Y | Y | Y | Y | N |
| **CXCL1_HUMAN** | N | N | N | N | N | N | N | N | N | N |
| **CXCL2_HUMAN** | N | N | N | N | N | N | N | N | N | N |
| **CXCL3_HUMAN** | N | N | N | N | N | N | N | N | N | N |
| **CXCL4_HUMAN** | N | N | N | N | N | N | N | N | N | N |
| **CXCL5A_HUMAN** | N | N | N | N | N | N | N | N | N | N |
| **CXCL5B_HUMAN** | N | N | N | N | N | N | N | N | N | N |
| **CXCL6_HUMAN** | N | N | N | N | N | N | N | N | N | N |
| **CXCL7_HUMAN** | N | N | N | N | N | N | N | N | N | N |
| **CXCL8_HUMAN** | N | N | N | N | N | N | N | N | N | N |
| **CXCL9_HUMAN** | N | N | N | N | N | N | N | N | N | N |
| **CXCL10_HUMAN** | N | N | N | N | N | N | N | N | N | N |
| **CXCL11_HUMAN** | N | N | N | N | N | N | N | N | N | N |
| **CXCL12a_HUMAN** | N | N | N | N | N | N | N | N | N | N |
| **CXCL12b_HUMAN** | N | N | N | N | N | N | N | N | N | N |
| **CXCL13_HUMAN** | N | N | N | N | N | N | N | N | N | N |
| **CXCL14_HUMAN** | N | N | N | N | N | N | N | N | N | N |
| **CXCL16_HUMAN** | * | * | * | * | * | * | * | * | * | * |
| **CX3CL1_HUMAN** | N | N | N | N | N | N | N | N | N | N |
| **XCL1_HUMAN** | N | N | N | N | N | N | N | N | N | N |
|  |  |  |  |  |  |  |  |  |  |  |
| * - Not tested as directly binding sensor |  |  |  |  |  |  |  |  |  |  |
| Y - binding detected at 300 nM, N = binding not detected |  |  |  |  |  |  |  |  |  |  |

| **Supplementary Table S2: Binding of evasins with mouse chemokines at 300 nM using biolayer interferometry.** | | | | | | | | | | |
| --- | --- | --- | --- | --- | --- | --- | --- | --- | --- | --- |
| **Chemokine** | **P991_AMBCA** | **P985_AMBMA** | **P546_AMBCA** | **P974_AMBCA** | **P983_AMBPA** | **P1181_AMBMA** | **P1182_AMBMA** | **P1183_AMBTR** | **P1180_AMBTR** | **P467_RHIPU** |
| **CCL2_MOUSE** | Y | N | N | N | Y | N | N | N | N | Y |
| **CCL3_MOUSE** | Y | Y | Y | Y | Y | Y | Y | Y | Y | Y |
| **CCL4_MOUSE** | Y | Y | Y | Y | Y | Y | Y | Y | Y | Y |
| **CCL5_MOUSE** | Y | Y | N | N | N | N | N | N | N | Y |
| **CCL6_MOUSE** | Y | Y | Y | Y | Y | N | N | N | N | Y |
| **CCL8_MOUSE** | Y | Y | Y | Y | Y | Y | Y | Y | Y | Y |
| **CCL9_MOUSE** | Y | N | N | N | N | N | N | N | N | Y |
| **CCL11_MOUSE** | Y | Y | Y | Y | Y | Y | Y | Y | Y | Y |
| **CCL12_MOUSE** | Y | Y | Y | Y | Y | Y | Y | Y | Y | Y |
| **CCL19_MOUSE** | Y | N | N | N | N | N | N | N | N | N |
| **CCL22_MOUSE** | Y | N | Y | Y | N | N | N | N | N | Y |
| **CCL24_MOUSE** | Y | N | Y | Y | N | N | N | N | N | Y |
| **CCL27_MOUSE** | Y | N | N | N | N | N | N | N | N | Y |
| **CCL28_MOUSE** | * | * | * | * | * | * | * | * | * | * |
|  |  |  |  |  |  |  |  |  |  |  |
| * - Not tested as directly binding sensor |  |  |  |  |  |  |  |  |  |  |
| Y - binding detected at 300 nM, N = binding not detected |  |  |  |  |  |  |  |  |  |  |

| **Supplementary Table S3.**  EC50 and EC80 values in Moles/Litre, mean, standard deviation (SD) and number of experiments (n), determined THP-1 cell migration assay | | | | |  |
| --- | --- | --- | --- | --- | --- |
| **Chemokine** | **MeanEC50** | **SDEC50** | **MeanEC80** | **SDEC80** | **n** |
| **CCL2_HUMAN** | 4.1E-10 | 3.99166E-10 | 1.0325E-09 | 1.06321E-09 | 4 |
| **CCL3L1_HUMAN** | 4.46667E-10 | 2.62742E-10 | 5.5E-10 | 9.16515E-11 | 3 |
| **CCL5_HUMAN** | 1.845E-09 | 3.8553E-10 | 8.6E-09 | 5.28583E-09 | 4 |
| **CCL7_HUMAN** | 2.35E-09 | 5.84038E-10 | 7.18E-09 | 3.37538E-09 | 3 |
| **CCL8_HUMAN** | 2.605E-09 | 1.33907E-09 | 7.265E-09 | 3.1079E-09 | 3 |
